# St. George’s Hospital

**Published:** 1858-12

**Authors:** 


[*London Medical Times & Gazette.*]

---

*St. George's Hospital.—Gallic Acid in Fungous Hæmatodes.*—(Under the care of Mr. TATUM.)—Case and remarks by Mr. C. HUNTER, House Surgeon.

The following is an interesting instance of the effect of an internal remedy on a malignant growth:—

On April 3, 1858, W. W. aged 8 years, was admitted in St. George's Hospital under the care of Mr. Tatum, with a small tumor not larger than the eye itself, and situated behind it in the left orbit; the eye consequently being protruded.

The tumor had attained this size in but two or three weeks; a little prominence of the upper eyelid had been however observed for four months.

Owing to the situation (behind the eye) and the rapid growth, no operative measures were had recourse to : the boy was however kept in the hospital, as the tumor rapidly increased.

As it grew larger, the eye, being pushed before it, gradually dwindled, and became at last a shrivelled-up and hardend excrescence on the outer part of the protruding mass.

In the course of four months (from time of admission) the tumor had become as large as the head of a 7 months fœtus, and of such a size as to overlap the mouth (so that he had to be fed by a pipe at the further corner of it.)

At this period (beginning of August), the surface of the tumor was irregu ar but rounded, the greater partof the surface was in a raw ulcerated condition, exceedingly vascular and constantly bleeding, often to such an extent that every attack appeared likely to be the last.

These hæmorrhagic attacks were generally treated by cold, by pressure, and by the local application of the blue lint. The boy was living on generous diet and wine. On the 2nd of august, after one of these attacks more serious than usual, which quite bleeched the face, and weakened the pulse (always weak and rapid), I gave him gallic acid in four grain doses, in infusion of bark, to try, if possible, to arrest the bleeding.

August 30, one month afterwards.—Curious as it may appear, the gallic aicd had been productive of the most marked effect, the tumor from that time had never bled once, nor even had there been the least oozing of blood. The surface of the mass became more healthy, less vascular, more solid, and *considerable diminution* of the tumor had taken place. After this, for a few days, increase of the tumor again occurred, but no bleeding took place from it. The increase in size was met with an increased dose of the gallic acid, which was again productive of benefit.

*Present State.*—Sept. 25.—1st. The tumor is about 6 inches measured over the longest diameter, and 8¼ over the shortest; this is much less than it was two months ago, so that the boy can now feed himself easily, the mouth not being at all overlapped, whereas before, he required feeding. 2nd. Not the least bleeding has occured since the first dose of the gallic acid, which was given now nearly two months ago. 3rd. The health, strength, and appetite of the boy appeared improved.

In recording this case it is only meant as an instance of the palliative effect of a remedy on malignant disease; it is the more curious that the gallic acid has had the striking effect it had, because of the exceedingly vascular and raw state of the surface. The least movement, the least cry used, before the administration of the acid, occasioned a sudden rush of blood from several parts of the tumour. That the tumor should have decreased in size is not less remarkable, than that all hæmorrhage for so long a time should have ceased.—*London Medical Times & Gazette.*

